# Disrupted TGF-**β** signaling: a link between bronchopulmonary dysplasia and alveolar type 1 cells

**DOI:** 10.1172/JCI178562

**Published:** 2024-03-15

**Authors:** Rongbo Li

**Affiliations:** Department of Pediatrics, University of California, San Diego, La Jolla, California, USA.

## Abstract

Bronchopulmonary dysplasia (BPD) is a chronic lung disease common in extreme preterm infants and is characterized by alveolar simplification. Current BPD research mainly focuses on alveolar type 2 (AT2) cells, myofibroblasts, and the endothelium. However, a notable gap exists in the involvement of AT1 cells, which constitute a majority of the alveolar surface area. In this issue of the *JCI*, Callaway and colleagues explored the role of TGF-β signaling in AT1 cells for managing the AT1-to-AT2 transition and its involvement in the integration of mechanical forces with the pulmonary matrisome during development. The findings implicate AT1 cells in the pathogenesis of BPD.

## AT1 cells in bronchopulmonary dysplasia

Extreme premature newborns experiencing respiratory distress often need supplemental oxygen and mechanical ventilation, putting them at a high risk of developing bronchopulmonary dysplasia (BPD), characterized by alveolar simplification and disrupted vascular development. Despite advancements in prenatal care improving BPD symptoms, patients often require prolonged ventilatory support and face an elevated risk of subsequent respiratory illnesses, including viral infections (1). Alveolar type 1 (AT1) cells cover over 90% of the alveolar surface area and play a crucial role in efficient gas exchange, owing to their squamous epithelial architecture and close association with the lung microvasculature. However, the precise mechanisms underlying BPD cellular defects, specifically linked to AT1 cells, remain incompletely understood.

In this issue of the *JCI*, Callaway and colleagues employed a mouse model with an AT1 cell–specific deletion of *Tgfbr2* (2). They found that the disruption of TGF-β signaling within AT1 cells during late lung development triggered a cell fate shift from AT1 to AT2 cells, leading to a BPD-like phenotype marked by impaired alveolarization and increased septal thickness. Additionally, they compared the impact of *Tgfbr2* loss in AT1 and AT2 cells by inactivating the gene in AT2 cells utilizing the *Sftpc-cre^ERT2^* driver. Interestingly, unlike the deletion in AT1 cells, mutant lungs lacking *Tgfbr2* in AT2 cells exhibited normal AT2-to-AT1 differentiation during development, suggesting that AT2 cells lacking *Tgfbr2* did not reprogram during late lung development. However, there was increased AT2 cell proliferation at P5, which led to an increased but nominal proportion of AT2 cells and a minor shift in the ratio of AT2 cells to AT1 cells in *Sftpc-cre^ERT2^:Tgfbr2* mutant lungs.

Subsequently, Callaway and colleagues utilized a mouse model with oligohydramnios, induced by prenatal reduction of amniotic fluid. This condition resulted in an increased AT1-to-AT2 cell-fate conversion. While the mouse oligohydramnios model and the AT1-specific *Tgfbr2* model exhibited similar phenotypes in regard to AT1-to-AT2 cell reprogramming, it appears that the oligohydramnios model may act through distinct mechanisms. This observation arises from the lack of difference between the oligohydramnios treated control and the *Hopx-cre^ERT2^:Tgfbr2* mutant in terms of AT1-to-AT2 cell reprogramming and mean septal thickness. Oligohydramnios induces a reduction in intraluminal stretch, while loss of *Tgfbr2* reduces the capacity of AT1 cells to respond to mechanical cues, leading to conversion towards the AT2 cell phenotype. This study is the first to our knowledge that utilizes AT1 lineage tracing to study oligohydramnios in the context of BPD compared with the previous oligohydramnios mouse model (3). The Callaway et al. findings align with those in their prior publication, which demonstrated that biophysical forces actively preserve AT1 cell identity through Cdc42 and Ptk2-mediated actin remodeling and cytoskeletal strain (4).

## The AT1 matrisome in development

To decipher the underlying molecular mechanisms, Callaway and colleagues conducted bulk RNA-Seq and proteomic analyses on neonatal AT1 cells isolated from *Hopx-cre^ERT2^:Tgfbr2* mutant mice and their heterozygous littermates. The data revealed that loss of *Tgfbr2* in AT1 cells led to downregulation of several genes associated with the lung matrisome. The lung matrisome, or lung extracellular matrix (ECM), is composed of collagens, elastin, proteoglycans and glycoproteins. Together, they provide the structural scaffolding for cells and confer the mechanical stability and elastic recoil crucial for proper lung function. Previous research has established that irregularities in the lung matrisome alter lung development and contribute to the progression of conditions like BPD. For instance, in a BPD mouse model induced by lipopolysaccharide (LPS) treatment coupled with hyperoxia, a reduction in fibronectin levels was observed in the lungs (5). Injection of a fibronectin inhibitor arrested alveolar development, suggesting a direct correlation between decreased fibronectin levels and the onset of BPD. Using a single-cell RNA-Seq dataset of the developing mouse lung previously generated by the group (6), Callaway and colleagues compared AT1 cell-enriched lung matrisome components across various development stages. They discovered that genes related to the matrisome exhibit high expression in AT1 cells, which started at E17.5 and remained elevated throughout adulthood. This finding underscores the lifelong importance of AT1 cells as a source for lung matrisome. To validate the findings from bulk RNA-Seq and proteomic analyses, which indicated a downregulation of lung matrisome genes upon *Tgfbr2* loss, researchers performed RNA scope and qRT-PCR assays on select lung matrisome genes expressed in AT1 cells. These experiments confirmed decreased expression levels of these genes in *Tgfbr2*-deficient mice when compared with controls.

To broaden the investigation beyond in vivo observations, researchers isolated AT1 cells from neonatal mice and cultured them in vitro with recombinant TGF-β or the TGF-β inhibitor SB431542. They measured the expression of AT1 ECM components and integrins, which were identified in the in vivo loss-of-function studies. Treatment with TGF-β1 elevated the expression of both *Itga5* and *Itgb1* compared with the control, whereas groups treated with SB431542 exhibited decreased expression of these integrins. Integrin-mediated cell-ECM interactions are key regulators of cellular behavior, impacting aspects such as spreading, motility, morphology, survival, proliferation, and differentiation. Indeed, AT1 cells obtained from *Hopx-cre^ERT2^:Tgfbr2* mutant animals displayed notably reduced spreading capacity compared with control cells. Similarly, AT1 cells treated with recombinant TGF-β exhibited a more spread-out appearance compared to the control group, whereas cells treated with SB431542 displayed reduced spreading. These findings indicate that TGF-β acts through regulating the expression of integrins and ECM components in AT1 cells to control AT1 cellular behavior, morphology, and function.

## Next steps and conclusions

Single-cell RNA-Seq has emerged as a powerful tool for mapping the transcriptomic landscape of individual cells, providing insights into cellular heterogeneity during lung development and disease. Recent applications of this approach have been instrumental in mapping cell populations within mouse models of hyperoxia-induced BPD (7–9). Findings from these studies highlighted the importance of cellular crosstalk and inflammatory signaling as key drivers of lung injury induced by hyperoxia. Data from single-cell transcriptomic analyses of human BPD lungs will provide further insights into disease pathogenesis and cellular responses. Future research directions should prioritize conducting single-cell transcriptomic analyses in BPD patients. Comparing these findings between human patients and mouse models will provide invaluable insights into potential species-specific differences in disease pathogenesis and cellular responses. The ultimate goal is to unravel the molecular mechanisms underpinning BPD, leading to the development of targeted therapeutic strategies for this devastating disease.

The lung is a complex structure comprising over 35 distinct cell types (10). Active interactions between AT1 cells and other resident lung cell types, such as mesenchymal and endothelial cells, are critical for alveolar development. For instance, AT1 cells predominantly express vascular endothelial growth factor A (VEGFA) (11), and studies have consistently reported lower VEGFA concentrations in infants who later developed BPD (12, 13). Similarly, platelet-derived growth factor subunit A (PDGFA) is primarily secreted by AT1 cells, and reduced PDGFRA expression has been reported in patients with BPD (14). Deletion of PDGFA/PDGFRA signaling in mice leads to simplified alveolar structures (15). In summary, these findings highlight the pivotal role of AT1 cells, acting as a source of ECM that is regulated by TGF-β, in orchestrating lung development. It opens the door to future investigations into the precise mechanisms by which the loss of TGF-β signaling in AT1 cells affects neighboring cell populations. Understanding these intricate intercellular interactions will shed light on how alterations in AT1 cells influence the development and progression of lung pathologies.

## Version 1 03/15/2024

Electronic publication
